# Experimental Mindfulness Intervention in an Emergency Department for Stress Management and Development of Positive Working Environment

**DOI:** 10.3390/healthcare11060879

**Published:** 2023-03-17

**Authors:** Alexandros Argyriadis, Louiza Ioannidou, Ioannis Dimitrakopoulos, Maritsa Gourni, Georgia Ntimeri, Chrisi Vlachou, Agathi Argyriadi

**Affiliations:** 1 School of Health Sciences, Frederick University, Nicosia 1036, Cyprus; 2 School of Education and Social Sciences, Frederick University, Nicosia 1036, Cyprus; 3 Hellenic Ministry of Health, Aristotelous 17, 10187 Athens, Greece

**Keywords:** mindfulness, stress management, well-being, health psychology, positive working environment, experimental intervention

## Abstract

Mindfulness-based interventions have increasingly gained the interest of health professionals in the last decade, especially practices that are short, economical, easily accessible, and physically, cognitively, and psychologically compelling. Nurses of Emergency Departments are a special, dynamic, but at the same time vulnerable group of health professionals who work in shifts and face multiple challenges. Considering the recent literature and the fact that stress and a hostile work environment are the top ranked health professionals’ challenges, there is a need for a further study of the use of mindfulness. This study aimed to investigate the effect of the application of mindfulness on nurses in the Emergency Department on several factors related to daily nursing practice and that directly affect these specific health professionals. This experimental study was performed on 14 participating nurses in the Emergency Department of a Public General Hospital in Athens, randomized into two groups: a control and an intervention group. The data collection tools were two digital smart devices, participatory observation, and semi-structured interviews. By practicing mindfulness meditation, the participating nurses in the intervention group showed improvement in their cognitive functions (attention, thinking, memory, concentration), professional interpersonal relationships, personal satisfaction and communication with patients and caregivers, sleep rate, negative emotions, and behaviors. The findings suggest that the application of mindfulness practices should be considered an easy, affordable, economical, accessible, and effective method that nurses can use to strengthen and empower themselves, enjoying its multiple benefits. The effectiveness of the application of mindfulness remains an important issue for future research in other health professionals as well.

## 1. Introduction

According to recent research, the modern demanding lifestyle directly impacts health and well-being, with stress being a global public health problem with negative psychosomatic consequences [1]. Several methods are used to deal with the adverse effects of the modern lifestyle on mental health, one of them being mindfulness. In the early 21st century, the application of mindfulness caused an explosion of interest and excitement, influencing medicine, psychology, politics, education, and even the media, appearing as a health “trend” on the radio, television, and in mainstream and academic journals [2].

Mindfulness describes a state of consciousness in which individuals observe and experience events in the present in a receptive and non-judgmental way. In 1990, John Kabat-Zinn first developed a mindfulness-based stress reduction program, the Mindfulness-Based Stress Reduction Program (MBSR). Because of the increased interest in this program, programs based on MBSR were designed to cultivate mindfulness and integrate its practice into daily life [3,4,5].

The implementation of these programs, in the majority of them, had a positive effect on the reduction and management of stress and improved and promoted the health and well-being of individuals both in the general population and in clinical practice [1,2,4]. Mindfulness-based interventions have increased interest among health workers who are exposed to emotional and physical vulnerability situations [5,6,7].

According to Xu et al. [8], nurses working in the Emergency Department face unique stressors that differ from those in other specialties. In particular, emergency nurses must make decisions for patients in acute situations, deal with a wide range of illnesses and injuries, and operate professionally, alertly, and flexibly in life-threatening situations while dealing with constant distractions or interruptions. Many mindfulness-based interventions for health professionals have been implemented, mainly in the USA [9,10,11,12,13], Brazil [14], Australia [15], Japan [16], Thailand [17], Malaysia [18], and Taiwan [19]. However, there is less data in Europe; for instance, some programs have been implemented in the UK [20] and Spain [21]. These programs indicated a significant reduction in health professionals’ stress and anxiety, empowering their well-being and mental health. Moreover, short mindfulness interventions using mobile applications in the health field highlight effective results at an individual (reducing stress and exhaustion) or organizational level (increasing patient satisfaction and reducing errors) [10,11].

## 2. Materials and Methods

Considering the literature findings, there emerges the need to develop and implement a short and affordable program that could support the psychological health professionals of the Emergency Department, as they work in a highly stressful environment. Therefore, the primary purpose of the current study was to develop and implement short mindfulness meditation practices for Emergency Department nurses, aiming to reduce stress and burnout. Given that no such interventions have been carried out in Greece, the present experimental intervention fills this gap.

In particular, this research examined whether: (a) work stress management is more effective after applying mindfulness sessions, (b) the patients’ satisfaction with the provided nursing health care services increased after the mindfulness training, (c) there was an improvement in the interpersonal relationships of the nurses, and (d) there was an improvement in the relationship between nurses, patients, and attendants. For these reasons, and in order to achieve the purposes of the study, we measured the heartbeats, blood pressure, and quality of sleep of the participants using several means, such as with smartwatches, interviews, and observation.

The main research questions of the study were:

1. Does mindfulness practice’s application positively impact emergency department nurses’ negative emotions?
2. Does mindfulness application affect nurse and patient satisfaction?
3. After applying mindfulness practices, is there a better organization and execution of professional tasks?
4. Does the application of mindfulness practices positively affect the sleep of healthcare professionals working in intensive care units?

### 2.1. Participants

The sample consisted of 14 nurses from the Emergency Department of a public general hospital of Athens (adults). The participants were categorized randomly into two groups: a control group and an intervention group. The sample was selected using the convenience sampling method because it was easy to access the sample (the researcher and participants work in the exact location of the study).

Regarding the gender of the participants, 14.3% (*n* = 2) were men and 85.7% (*n* = 12) were women. Of the participants, 42.8% were between 30–39 years old, 28.6% were between 18–29 years old, 14.3% were 40–49 years old, and 7.1% were 50–59 years old. Regarding marital status, 57.2% were singles (*n* = 8), 28.6% were married (*n* = 4), 7.1% were divorced (*n* = 1), and 7.1% were widowhood (*n* = 1). The majority of participants declared 0 children (57.2%), followed by 2 children (28.6%) and 1 child (14.2%). Half of the nurses, with a percentage of 50%, declared a TEI graduation diploma, 21.4% had a lower graduation diploma, 14.3% had a university diploma, and 14.3% graduated from another school related to nursing. Regarding experience in years, the majority, with a percentage of 57.2%, had up to 10 years of experience, 21.4% had work experience between 10–20 years, 7/1% had work experience between 20–30 years, and 14.3% had more than 30 years of experience. All the nurses were Greek (100%). Finally, 92.9% of the participants stated that they had no experience with mindfulness, while only 1 person stated a previous experience (7.1%) about a year before participating in this research.

### 2.2. Procedure

For the present research purpose, the researcher designed the thematic sections and questions of the semi-structured interview and the observation characteristics. In addition, only participants in the intervention group participated in mindfulness meditation practices, including breathing. Data were collected through a smartwatch device, smartphone application, participant observation, and semi-structured interviews. Data included the effect of mindfulness practices on negative emotions, satisfaction, organization and performance of professional tasks, and the sleep of Emergency Department nurses.

Before the start of the study, a meeting was arranged for all participants with the researcher. Participants brought their smartphones with them at this preparatory meeting to install the mobile application. Following this, participating nurses in the intervention group completed a ten-day data recording program which included measurements related to blood pressure (measurement before lying down and standing up), heartbeats (maximum, minimum, and average heartbeats), and sleep status (deep and light sleep in %). The nurses wore the smartwatch on their wrists throughout the intervention and filled in the requested measurable data from their mobile application (the smartwatch data were recorded via the mobile device application). The start date was 28 March 2022, and the end date was 6 April 2022. A baseline measurement before the intervention was also taken for each participant.

For the analysis of the data from smartwatches, descriptive statistics with the use of the SPSS statistical program were used. For the results coming from interviews and observation, the thematic content analysis took place.

Frederick University’s Bioethics Committee has approved the research work protocol. In addition, before the study implementation, participants gave their written consent and were informed about the purpose of the research.

## 3. Results

### 3.1. Results from the Data Recording for Ten Consecutive Days

#### 3.1.1. Systolic and Diastolic Blood Pressure

In the present study, a ten-day recording of the systolic and diastolic pressure upon standing and before lying down was performed on all intervention and control participants. In the intervention group, the systolic pressure before lying down varied between 107–112 mmHg with an average of 111 mmHg, and the diastolic pressure varied between 50–68 mmHg with an average of 62 mmHg. In the control group the systolic pre-bed pressure ranged from 115–129 mmHg with an average of 112 mmHg, and the diastolic from 68–74 mmHg with an average of 72 mmHg. Regarding the standing blood pressure for the intervention group, the systolic pressure ranged from 103–110 mmHg with an average of 106 mmHg, and the diastolic from 57–63 mmHg with an average of 60 mmHg. On the other hand, for the control group, the standing systolic pressure ranged from 108–117 mmHg with an average of 112 mmHg, and the diastolic pressure from 63–72 mmHg with an average of 60 mmHg.

#### 3.1.2. Heartbeats

Regarding heartbeats, we recorded the maximum, minimum, and average number of heartbeats per recording day. Specifically, in the intervention group, the maximum number of heartbeats ranged from 97–109 bpm, with average maximum heartbeats of 102 bpm. The minimum number of heartbeats was between 48–54 bpm, with an average of minimum heartbeats of 52 bpm, while the overall average was 72 bpm. In the control group the maximum number ranged from 94–112 bpm with average maximum heartbeats of 100 bpm. On the other hand, the minimum number of heartbeats was between 59–63 bpm with an average of minimum heartbeats of 61 bpm, while the overall average was 74 bpm.

#### 3.1.3. State of Sleep

Regarding the state of sleep in the intervention group, the deep sleep ranged between 27.6–41.7% with an average of 33.8%. The light sleep ranged from 58.3–72.4% with an average of 66.2%. In the control group the percentage of deep sleep was between 27.1–34% with an average of 29.1%, and the light sleep ranged from 66–72.2% with an average of 70.9%.

Intervention Group (Ten Day Tables) (Table 1, Table 2 and Table 3).

Control Group (Ten Day Tables) (Table 4, Table 5 and Table 6).

### 3.2. Results from the Participatory Observation

#### 3.2.1. Intervention Group Results

Initially, it was found that participants indicated negative emotions mostly on days of general duty when the hospital receives a large number of cases. The main negative feeling was the frustration due to the lack of staff, the poor logistical infrastructure of the department, and the administrative inadequacy.

Regarding attention ability, observation results highlighted that participants maintained high positive levels of attention during their work, a fact significant for the proper operation of the department and the proper management of incidents. In addition, especially after the 6th–7th day, it was observed that the nurses’ attention was not easily distracted and that they were more focused on what they did, although there were situations that could easily distract attention, such as stimulating incidents and tensions between patients. Moreover, the tension and anger levels gradually decreased, which have been affected by mindfulness meditations.

Regarding the conflicts between nurses, it was observed that they were kept at low levels, a positive fact for the orderly operation of the department. However, the voice pitch decreased after the fourth day of observation. It was recorded that the lower the tone of the nurses’ voice was, the lower the tension was between the interdisciplinary team and the patients and their companions.

Regarding the smile and humor of the staff, it was observed that they were improved gradually, a fact that possibly affected both personal and patient satisfaction.

Regarding the satisfaction of staff and patients, it was recorded that the levels differed per day. Environmental factors that influenced the satisfaction level were the quality and number of patients and attendants, professional fatigue, the reduction of tension and anger, and the increased positive emotions of the nurses.

#### 3.2.2. Control Group Results

Regarding the control group, negative emotions predominate on all days but much more on general duty days. This group was dominated by disappointment and dissatisfaction due to the exact causes mentioned above in the experimental group.

Regarding intensity, tone of voice, and anger, it was recorded that they ranged from moderate to high levels. This affected their interpersonal and interprofessional relationships, their personal satisfaction, and the satisfaction of the patients and attendants. More generally, this attitude and behavior led to a negative working climate which affected the department’s orderly operation, with inevitable conflicts.

Smiling and humor ranged from low to moderate levels, while efficiency and attention ranged at high levels. Furthermore, the nurses refused to work mainly on the day of general duty and the following day, indicating increased anxiety levels without affecting their attention and work performance.

### 3.3. Results from the Interviews in the Intervention Group

Below are the most indicative answers of the participants:

Participants in the intervention group reported feelings of relaxation, calmness, less stress, and relief after the mindfulness meditations. Indicatively, a nurse mentioned: “I felt calmer after each session”. Other nurses claimed: “Relieved from anxiety and calmer”, “I feel relief”.

Regarding the changes they noticed in themselves after the meditations, they reported a generally positive effect, clearer thinking, reduced nervousness, improved performance, and less anxiety. Specifically, they reported: “I noticed that my concentration increased and I performed better in many areas of my daily life”, “I was calmer and less nervous”, “I was definitely calmer and less anxious”. However, a nurse’s response was: “I haven’t noticed anything yet, maybe because the experience was too short”.

Whether mindfulness meditations positively or negatively affected the attention, concentration, and memory of Emergency Department nurses, the majority of participants reported a positive effect. Furthermore, participants stated that anxiety levels decreased after the meditations. In particular, they mentioned “It was greatly affected. I wasn’t that stressed. I was calmer and more relaxed,” “Yes. I felt less stressed. I noticed that when a severe traffic accident happened on one of my shifts, I was much calmer, focused, and less stressed, and I was not distracted by the surrounding voices and the panic that prevailed”.

Regarding the average amount of sleep per day, most nurses reported: “from 4 to 7 h”. However, one nurse reported, “There are days I might sleep only 1 or 2 h. I have to take care of my family too. Sleep for me is a luxury”, and another nurse stated, “Some days, especially after a night shift, I find it difficult to sleep“.

In a subsequent question about how they sleep after mindfulness meditations, two nurses answered: “I didn’t notice a difference” and some others that, “My sleep is lighter”, and “I sleep a little more immediately”. Additionally, a nurse mentioned that “I fall asleep faster without my mind wandering into worries and concerns” and another that “I sleep much calmer and relaxed without waking up and being overexcited”.

Regarding having a different feeling when they wake up after the meditations, five of the seven nurses reported waking up more rested and having a pleasant and optimistic mood. Another nurse mentioned that “When I wake up my mood is low, and I think that’s because I need some more time” while another nurse said “No, I’m facing the new day”.

When participants were asked about their professional interpersonal relationships after the meditations, most responses were positively related. Expressly, the following responses were noted: “Yes, I noticed that I had a calmer and better aura”, “After the meditations, I noticed that I continued my work with more interest”, “They were definitely affected since to a large extent I can control my emotions”, “ I have fewer nerves”, “I am more patient”, and “ I could better manage my stress, anger, and tension, I felt calmer, so I had better communication and cooperation with my colleagues”.

The majority of responses were negative in the question of whether mindfulness practices affected the organization of daily nursing tasks. However, the positive answers were the following: “There was a difference because I started with clearer thinking, so everything went more correctly”, “I had an energy and a positive attitude,” and “I noticed a calmer and more focused attitude in the organization of my work”. The negative answers that prevailed were: “No”, “I think not” and “No, I did not notice a difference”.

## 4. Discussion

The nature of the work of nurses working in the Emergency Department is recognized as particularly stressful [18] due to excessive workloads, administrative tasks, time constraints, tense relationships and interprofessional conflicts, the numerical inadequacy of colleagues, exhausting shifts, and suffering and caring for patients with life-threatening illnesses [1,22,23]. Based on all those mentioned above, the nurses are led to physical and psychological exhaustion with the risk of developing diseases (mainly cardiovascular diseases), threatening their lives and even sudden death [24].

Research results showed that the average standing blood pressure for the control group was 112/60 mmHg and for the intervention group it was 106/60 mmHg, while the blood pressure before lying down for the control group was 112/72 and for the intervention group it was 111/62. There appears to be a small deviation between the groups’ results in relation to the blood measurements. According to previous studies, the change in pressures and even small changes that are not clinically significant demonstrate that mindfulness practices are associated with a reduction in blood pressure that contributes to a lower risk of cardiovascular diseases [25,26,27].

Regarding the results of the heartbeats, the control group’s average maximum heartbeats were 100 bpm, the minimum were 61 bpm, and the overall average was 74 bpm. For the intervention group, the average maximum for the heartbeats was 102 bpm, the minimum 52 bpm, and the overall average was 72 bpm. In line with these results, Entus’s study showed that mindfulness intervention contributed positively to heart rate reduction, although there was a slight variance in values.

Regarding the sleep status results, the average sleep of the participants ranged from 4 to 7 h. The percentage for the control group in the ten-day recording related to light sleep was 70.9% and for deep sleep it was 29.1%, while for the intervention group the average for light sleep was 66.2% and for deep sleep it was 33.8%. It appears that the experimental group was positively affected by the application of mindfulness meditations since the percentage corresponding to deep sleep was higher, by 4.7%. The difference in deep sleep in favor of the intervention group indicates the positive effect of mindfulness meditations on deep restorative sleep. A randomized controlled trial that used a “personal anchor” to create a meditation practice routine with a mobile phone app argued that mindfulness meditation could also positively affect sleep [28,29].

Anxiety levels and negative emotions, including frustration, were higher in the control group than in the intervention group, which is in line with the results of other studies that support mindfulness meditation’s positive effect on managing negative emotions and anxiety [2].

The tension during work, anger, conflicts, tone of voice, and interpersonal relations were at a moderate level for the participants in the intervention group, while for the participants in the control group they were at a high level. This finding suggests that mindfulness meditations positively affected the intervention group behavior. This finding is consistent with the conclusions from the National Database of Nursing Quality Indicators (NDNQI) and Practice Environment Scale (PES), indicating that mindfulness practice is an effective and accessible tool for improving job satisfaction, team collaboration, and creating a healthy work environment (Monroe et al., 2021). Additionally, in a review that investigated the benefits of mindfulness practices in psychotherapy, improvements in interprofessional relationships, attention, conflicts, and tensions in the workplace were highlighted [30,31]. In addition, the efficiency and attention of the participants of both groups varied at high and without significant deviation levels.

The results differed for the two groups regarding personal and patient satisfaction. The control group scored lower in terms of patient satisfaction, probably due to burnout, increased tension, anger, and conflict than in the intervention group. However, the nurses of the intervention group had a higher score in their personal and patient satisfaction than the control group, a fact that can be attributed to the effect of the mindfulness meditations.

The interview included four categories of questions to collect information related to the assessment of the participants’ experience of mindfulness meditations, sleep status, effects of meditations on professional employment, and experiences in relation to patients and their attendants. The majority had a positive effect regarding the evaluation of the experience and the changes observed by the participants from the guided and short ten-day mindfulness meditations. Specifically, they reported reduced nervousness and stress, relaxation, calmness, improved work performance, and “clearer” thinking. Regarding attention, concentration, and memory, most nurses claimed that meditation positively affected them. Specifically, they mentioned that there was a feeling that: “the mind is empty from the tension of the day”, which helped to improve attention, concentration, and memory. Others stated: “I would like to implement this practice more regularly as I noticed that my attention span was improved”. The application of mindfulness practices offers multiple benefits to cognitive functions such as attention, thinking, memory, and critical thinking, as supported by the literature and previous research findings [32,33]. However, only one nurse declared that he did not notice any effect on attention, concentration, and memory, perhaps because, as he mentioned, his experience with mindfulness meditations was brief. This opinion is likely because the mindfulness meditations were few (ten in total) and he needed more time to receive their benefits. This opinion may also be due to personal obstacles (such as impatience and lack of motivation).

Sleep is a normal state that plays a vital role in restoring fatigue [34]. Participating nurses reported, on average, that they sleep 4–7 h a day, while sometimes it can be 1 to 2 h per day, and this is because they all work alternating shifts. An adult usually needs an average of 7–9 h of sleep per day [35], and shift work seems to negatively affect the quantity and quality of sleep [35]. Regarding the quality and quantity of sleep and the feeling of waking, most nurses had a positive effect from the mindfulness meditations. They said they slept, woke up more calmly, and did not feel sleepy during the day.

## 5. Conclusions

The implementation of mindfulness practices has been shown to be an effective method for Emergency Department nurses, which has a positive effect on the management and creates a reduction in burnout and stress while providing them with multiple benefits at the cognitive, physical, mental, and emotional levels. This research highlighted the reality experienced by nurses in this department and, at the same time, their need to improve and eliminate the factors that create negative mental feelings, pressure, and excessive demands.

Mindfulness meditations can lead to the development of new approaches that will promote the physical and mental health and well-being of Emergency Department nurses, with an impact on the well-being and satisfaction of patients and their attendants with whom they interact. In addition, mindfulness can be widely applied to other health professionals by enhancing their well-being and improving the health services provided. Future research should investigate the long-term effects of mindfulness meditation on ED nurses and incorporate it into daily clinical practice. Additionally, future research applying mindfulness meditations to the administrative and nursing leadership of public hospitals may bring promising results by improving job performance and satisfaction, communication, and interprofessional and interpersonal relationships.

## 6. Limitations

Research limitations about practicing mindfulness in health professionals and hospitals are related to the low number of participants and the general lack of information about the effects of mindfulness on health. Nurses for example may not be motivated to practice mindfulness because of how little data exist on its actual benefits as well as due to lack of time. While some studies have found that mindfulness increases satisfaction and well-being among health professionals, more research is needed to understand the impact of mindfulness on health outcomes. Additionally, research limitations also include the complexity of the practice of mindfulness itself. Mindfulness is a complex and multifaceted concept that is best understood through personal practice. Furthermore, due to the subjective nature of mindfulness, it can be difficult to standardize the practice in a way that would be suitable for research. The lack of research on mindfulness in health professionals and hospitals also has implications for policy making and implementation. While some hospitals may be interested in incorporating mindfulness into their practices and policies, without sufficient evidence of its benefits, it is difficult to justify such decisions. Additionally, there is a lack of a consensus regarding the best ways to incorporate mindfulness into healthcare settings. Without a clear understanding of the effects of mindfulness, it is difficult to develop a policy or program that would effectively utilize the practice. In conclusion, there are a number of research limitations associated with practicing mindfulness in health professionals and hospitals. These include a lack of information about the effects of mindfulness, the complexity of the practice, and ethical considerations. As such, more research is needed to understand the effects of mindfulness on health outcomes and to develop effective policies for its implementation in healthcare settings. This section is not mandatory but may be added if there are patents resulting from the work reported in this manuscript.

## Figures and Tables

**Table 1:** Blood pressure results.

Blood Pressure	1st Day	2nd Day	3rd Day	4th Day	5th Day	6th Day	7th Day	8th Day	9th Day	10th Day
At rest	111/58	109/58	112/64	107/59	116/65	111/60	111/68	112/62	112/64	111/64
Waking up	103/59	107/59	106/62	104/60	106/61	106/58	107/59	109/58	105/57	110/63

**Table 2:** Heartbeat results.

Heartbeats	1st Day	2nd Day	3rd Day	4th Day	5th Day	6th Day	7th Day	8th Day	9th Day	10th Day
Max	98	100	109	101	105	97	105	102	100	100
Min	49	48	52	54	53	51	53	54	52	52
Average	70	72	74	70	72	70	73	72	69	74

**Table 3:** Sleep quality results.

Sleep	1st Day	2nd Day	3rd Day	4th Day	5th Day	6th Day	7th Day	8th Day	9th Day	10th Day
Deep Sleep%	28.1%	34.7%	34.6%	31.1%	38.1%	29.4%	41.7%	38.9%	33.7%	27.6%
Light Sleep%	71.9%	65.3%	65.4%	68.9%	61.9%	70.6%	58.3%	61.1%	66.3%	72.4%

**Table 4:** Blood pressure results.

Blood Pressure	1st Day	2nd Day	3rd Day	4th Day	5th Day	6th Day	7th Day	8th Day	9th Day	10th Day
At rest	125/74	121/68	123/74	116/70	116/73	126/72	115/72	123/69	118/72	129/72
Waking up	113/66	111/71	111/69	111/72	108/72	115/63	108/68	111/68	114/64	117/67

**Table 5:** Heartbeat results.

Heartbeats	1st Day	2nd Day	3rd Day	4th Day	5th Day	6th Day	7th Day	8th Day	9th Day	10th Day
Max	101	97	101	94	96	101	101	112	95	101
Min	60	61	60	60	59	63	61	61	62	62
Average	74	71	74	71	74	76	76	75	75	73

**Table 6:** Sleep quality results.

Sleep	1st Day	2nd Day	3rd Day	4th Day	5th Day	6th Day	7th Day	8th Day	9th Day	10th Day
Deep Sleep%	27.7%	28.3%	29.8%	34%	29%	30.3%	27.1%	27.9%	27.9%	28.9%
Light Sleep%	72.3%	71.7%	70.2%	66%	71%	69.7%	72.9%	72.1%	72.1%	71.1%

## Data Availability

The data presented in this study are available on request from the corresponding author.
